# Minimally Modified LDL Upregulates Endothelin Type A Receptors in Rat Coronary Arterial Smooth Muscle Cells

**DOI:** 10.1155/2013/656570

**Published:** 2013-06-19

**Authors:** Jie Li, Lei Cao, Cang-Bao Xu, Jun-Jie Wang, Yong-Xiao Cao

**Affiliations:** ^1^Department of Pharmacology, Xi'an Jiaotong University College of Medicine, Xi'an, Shaanxi 710061, China; ^2^Department of Pharmacy, The First People's Hospital of Chenzhou, Institute of Translational Medicine, Nanhua University, Chenzhou, Hunan 423000, China; ^3^Institute of Basic and Translational Medicine, Xi'an Medical University, Xi'an, Shaanxi 710021, China; ^4^Department of Pharmacology, Xiangnan University, Chenzhou, Hunan 423000, China

## Abstract

Minimally modified low-density lipoprotein (mmLDL) is a risk factor for cardiovascular disease. The present study investigated the effects of mmLDL on the expression of endothelin type A (ET_A_) receptors in coronary arteries. Rat coronary arteries were organ-cultured for 24 h. The contractile responses were recorded using a myographic system. ET_A_ receptor mRNA and protein expressions were determined using real-time PCR and western blotting, respectively. The results showed that organ-culturing in the presence of mmLDL enhanced the arterial contractility mediated by the ET_A_ receptor in a concentration-dependent and time-dependent manner. Culturing with mmLDL (10 **μ**g/mL) for 24 h shifted the concentration-contractile curves toward the left significantly with increased *E*_max_ of 228% ± 20% from control of 100% ± 10% and significantly increased ET_A_ receptor mRNA and protein levels. Inhibition of the protein kinase C, extracellular signal-related kinases 1 and 2 (ERK1/2), or NF-**κ**B activities significantly attenuated the effects of mmLDL. The c-Jun N-terminal kinase inhibitor or the p38 pathway inhibitor, however, had no such effects. The results indicate that mmLDL upregulates the ET_A_ receptors in rat coronary arterial smooth muscle cells mainly *via* activating protein kinase C, ERK1/2, and the downstream transcriptional factor, NF-**κ**B.

## 1. Introduction

Oxidized low-density lipoprotein (oxLDL) is not limited to atherosclerotic plaques but can circulate as minimally modified LDL (mmLDL) that is formed when only the lipid region of LDL is oxidized. mmLDL is a potential biomarker for cardiovascular disease. It enhances cytokine production and expression of CD14 and toll-like receptor, induces proinflammatory activities in monocytic cells [1], damages endothelial function, promotes the formation of oxLDL and foam cells, and enhances vascular cell migration and proliferation [2]. These effects contribute to atherosclerotic lesion formation [3], which occurs through a mechanism involving the stimulation of receptor-mediated signal transduction pathways [4]. 

Endothelin peptides are produced in the endothelium of vessels [5]. Endothelin-1 (ET-1) stimulates vascular smooth muscle cell proliferation [6], migration [7], contraction [8], matrix remodeling [9, 10], synthesis of extracellular matrix components [11], and the expression of other proatherogenic growth factors, such as platelet-derived growth factor and transforming growth factor-beta [12]. There are two types of endothelin receptors in the vasculature of mammals, the endothelin type A (ET_A_) and endothelin type B (ET_B_) receptors, which are involved in ischemic cardiovascular disease by enhancing the contraction and proliferation of smooth muscle cells [13]. The expression of ET-1 and its receptors is upregulated in experimental models of atherosclerosis and in human atherosclerotic lesions [14, 15]. 

We have developed an organ culture model that mimics the upregulation of receptors in cardiovascular disease [16, 17]. This organ culture allows in-depth investigation of the intracellular mechanisms underlying the alteration in the expression of the ET receptors in rat coronary arteries. Using this model, we have demonstrated that mmLDL upregulates ET_B_ receptors in both the rat coronary artery and basilar artery via activation of signal transduction pathways [18, 19]. 

The mitogen-activated protein kinases (MAPK) include the extracellular signal-regulated proteins 1 and 2 (ERK1/2), c-Jun N-terminal kinase (JNK), and the p38 cascade proteins [20] and play an important role in the intracellular signaling that occurs in response to extracellular stimuli [20], which causes phosphorylation and activation of transcription factors in the cytoplasm or the nucleus [21]. Protein kinase C (PKC) participates in signal transduction events in response to specific hormonal, neuronal, and growth factor stimuli [22]. NF-*κ*B is a pivotal transcription factor downstream of the MAPK and PKC pathways [18, 19]. Activation of NF-*κ*B is essential for controlling the inducible expression of several genes involved in inflammation and cell proliferation. 

It is well known that both mmLDL and ET_A_ receptors upregulation is involved in inflammation and the pathogenesis of atherosclerosis; however, their relationship is unclear. The present study was designed to investigate the hypothesis that mmLDL upregulates ET_A_ receptor in rat coronary arterial smooth muscle cells and the possible intracellular mechanisms.

## 2. Materials and Methods 

### 2.1. Reagents

mmLDL and LDL were obtained from the Xiehe Research Institute (Beijing, China). ET-1 and sarafotoxin 6c were purchased from Auspep, Parkville, Australia and dissolved in 0.9% saline with 0.1% bovine serum albumin. DMSO was used to dissolve staurosporine, SB386023, U0126, SP600125, SB203580, and wedelolactone (Sigma, St. Louis, MI, USA). BQ-788 (Sigma, St. Louis, MO, USA) was dissolved in 0.9% saline. Analytical grade chemicals and double-distilled water were used throughout the experiments. All of the drugs were further diluted in buffer solution immediately before being used in the experiments. The concentrations were expressed as the final molar concentration in the tissue baths. 

### 2.2. Animals

Three hundred and thirty Sprague-Dawley rats (300–350 g) were obtained from the Animal Center of Xi'an Jiaotong University College of Medicine, China, and handled according to the guidelines provided by the Animal Care and Use Committee at Shaanxi Province. The experimental protocols were approved by the animal ethics committee at Xi'an Jiaotong University.

### 2.3. Organ Culture of Coronary Arteries

Rats were anaesthetized with CO_2_ and decapitated to prepare artery samples. The hearts were removed and immersed into cold buffer solution. Under a dissection microscope, the left anterior descending coronary artery was gently excised from the myocardium [23, 24] and freed from the adhering tissue. The arteries were then cut into approximately 1-2 mm long ring segments. For organ culture, the coronary artery ring segments were placed in 24-well plates, two segments in each well containing 1 mL of Dulbecco's Modified Eagle's Medium [17]. The arterial segments were cultured with mmLDL (10 *μ*g/mL) or LDL (10 *μ*g/mL). An organ culture group was added as a control to eliminate the impact of organ culture *per se* on the experimental results. To examine the mechanism of the effects, the specific inhibitors of different signal transduction pathways were used. The inhibitors and mmLDL were added to Eagle's medium simultaneously at the beginning of the organ culture process.

In order to study the effect of the intracellular signaling pathways on the upregulation, we used some pathway inhibitors such as the PKC pathway inhibitor, staurosporine (0.1 *μ*M) [22]; the inhibitors selected to target the different kinases leading to ERK1/2 activation, U0126 (10 *μ*M) and SB386023 (10 *μ*M); the specific JNK and p38 MAPK inhibitors, SP600125 (10 *μ*M) and SB203580 (10 *μ*M) [18, 19]; the NF-*κ*B-inhibitor, wedelolactone (10 *μ*M) [25, 26]. Each inhibitor was present for 24 h. Thereafter, the artery segments were mounted in myography baths. For analysis by real-time PCR or western blotting, the vessels were frozen in liquid nitrogen for 3 h and then stored at −80°C until they were processed. 

### 2.4. Myographic Studies

The isometric tension in the isolated coronary arterial segments was recorded using a myography system [23, 24]. The artery segments were threaded on two 40 *μ*m diameter stainless steel wires and mounted in myography baths. One wire was connected to a force-displacement transducer attached to a computer. The other wire was attached to a movable displacement device allowing fine adjustments of the vascular tension. The arterial segments were immersed in baths containing Krebs solution (37°C) [27]. The contractile capacity was determined by exposure to a potassium-rich (63.5 mM) Krebs solution. The segments were used only if potassium elicited reproducible responses greater than 0.8 mN. Concentration-response curves were obtained by cumulative addition of the agonists to the baths. A specific ET_A_ receptor agonist was not found. To study ET_A_ receptor-mediated contraction, sarafotoxin 6c was added to the baths to a final concentration of 1 *μ*M to induce a contraction, and the segments remained in the sarafotoxin 6c (1 *μ*M) supplemented solution for an additional 1 h to desensitize the ET_B_ receptor. Concentration-response curves for the agonist ET-1 (an ET_A_ and ET_B_ receptor agonist) were obtained by cumulative application of the substance (10^−10^ M–10^−7^ M). During this period, the contractile response to sarafotoxin 6c faded to the baseline level even though sarafotoxin 6c was still present in the bath with the segments. After the ET_B_ receptors had been desensitized, the concentration-effect curve induced by ET-1 was obtained. Thus, the contractile response to ET-1 was mediated only by the ET_A_ receptors [28, 29]. To confirm the desensitization of the ET_B_ receptors, the effect of the selective ET_B_ receptor antagonist, BQ-788 (0.1 *μ*M), on the ET-1-induced contractions after sarafotoxin 6c desensitization was examined. The ET-1-induced contractions were similar in the presence and absence of BQ-788, suggesting the activation of only the ET_A_ receptors.

### 2.5. Real-Time PCR

A RNAfast200 Kit (Shanghai Flytech Biotechnology Co., Ltd., Shanghai, China) was used to extract the total RNA. The resulting pellet was washed with 75% ethanol, air-dried, and redissolved in 40 *μ*L of diethylpyrocarbonate-treated water. The OD_260_/OD_280_ ratios were between 1.9 and 2.1. The concentration of the total RNA was approximately 3 *μ*g/*μ*L. Reverse transcription of total RNA to obtain cDNA was conducted using a GeneAmp RNA PCR Kit (Applied Biosystems, Beijing, China) and a Perkin-Elmer DNA thermal cycler. First-strand cDNA was synthesized from total RNA in a 40 *μ*L reaction volume using random hexamers as primers. The reaction mixture was incubated at 25°C for 10 minutes, heated to 42°C for 15 minutes, heated further to 99°C for 5 minutes, and chilled to 5°C for 5 minutes. Real-time PCR was performed in a GeneAmp 5700 sequence detection system using the GeneAmp SYBR Green Kit (Toyobo Co., Ltd., Osaka, Japan) with the previously synthesized cDNA as the template in a 25 *μ*L reaction volume. A no-template control was included in all of the experiments. The primers were designed using Primer Express 2.0 software and were synthesized by Beijing Sunbiotech Co., Ltd. (Beijing, China). The specific primers for the rat ET_A_ receptor (GenBank accession number NM_012550) were as follows:

ET_A_ *receptor *

 forward: 5′-GCTCAACGCCACGACCAAG-3′
 reverse: 5′-GTGTTCGCTGAGGGCAATCC-3′.

The housekeeping gene *β*-actin (GenBank accession number NM_031144) was used as the internal control. The primers used were as follows:

**β*-actin*

 forward: 5′-ACTATCGGCAATGAGCGGTTCC-3′
 reverse: 5′-CTGTGTTGGCATAGAGGTCTTTACG-3′.

Real-time PCR was performed using the following profile: 95°C for 1 minute, followed by 40 cycles at 95°C for 15 seconds, 60°C for 15 seconds, and 72°C for 45 seconds. Dissociation curves were run after the real-time PCR was complete to identify the specific PCR products.

### 2.6. Western Blotting

Cultured or fresh coronary arterial segments were stored at −80°C. The total proteins were quantified using a BCA Protein Assay Kit (Pierce Biotechnology, Shanghai, China) according to the manufacturer's instructions, separated on SDS-PAGE gels, transferred to a polyvinylidene difluoride (PVDF) membrane, and blocked with 5% nonfat dry milk. The immunoblots were incubated with primary antibodies directed against endothelin receptor A (1 : 100) (Millipore, CA, USA). The immunolabeled protein bands were detected using Super Signal Chemiluminescent Substrate after incubation with horseradish peroxidase-conjugated secondary antibody (1 : 5000). *β*-actin was used as the internal loading control. Densitometric analysis was performed using Image Gauge verion 4.0 software (Fuji Photo Film Co., Ltd., Japan). 

### 2.7. Calculations and Statistics

The maximum contraction (*E*_max⁡_) value was calculated as the percentage of the contraction induced by 63.5 mM K^+^, and the pEC_50_ value refers to the negative logarithm of the molar concentration of a drug that produces half-*E*_max⁡_. The concentration-effect curve of each agonist was fitted to the Hill equation using an iterative, least square method (GraphPad Prism 5) to provide estimates of the *E*_max⁡_ and pEC_50_ values. All of the real-time PCR experiments were performed in duplicate, and the mean values were used. The amount of ET_A_ receptor mRNA was calculated relative to the level of the mRNA expression of the *β*-actin housekeeping gene in the same sample. The following formula was used to calculate the amount of ET_A_ receptor mRNA: *X*_0_/*R*_0_ = 2^*C*_*tR*_−*C*_*tX*_^, where *X*_0_ = the original amount of endothelin ET_A_ receptor mRNA, *R*_0_ = the original amount of *β*-actin mRNA, *C*_*tR*_ = the *C*_*t*_-value for *β*-actin, and *C*_*tX*_ = the *C*_*t*_-value for the ET_A_ receptor. The amount of receptor protein relative to the amount of the internal control is expressed as a percentage of the value for the control group. 

The statistical analyses of the myography experiments and real-time PCR experiments were based on one measurement per rat. When the number of arterial segments was more than one in an individual, the average was used for that individual in myograph experiment. In the Western blotting experiments, each sample was a pool of 4 coronary arterial segments. All of the data are expressed as the mean values ± SEM. Student's *t*-test was used to compare two sets of data, and a one-way analysis of variance (ANOVA) or a two-way ANOVA followed by Dunnett's test (GraphPad Prism) was used for comparisons of more than two data sets. A *P* value of less than 0.05 was considered significant. 

## 3. Results

### 3.1. Upregulation of ET_A_ Receptors in the Coronary Artery

**Figure 1:**
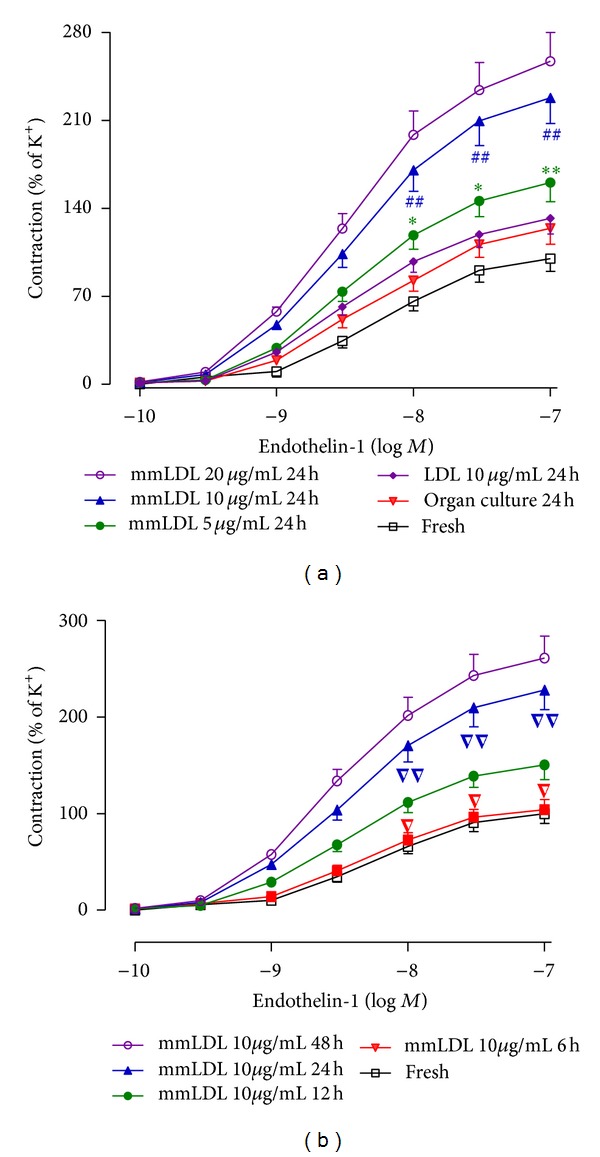
Culturing with mmLDL enhanced the ET-1-induced contraction of rat coronary artery segments. (a) The concentration-dependent effect of culturing with mmLDL for 24 h; *P < 0.05, **P < 0.01  *versus *organ culture for 24 h; ^##^ *P* < 0.01  *versus* 5 *μ*g/mL of mmLDL for 24 h. (b) The time-dependent effect of culturing with 10 *μ*g/mL of mmLDL. ^▽^ *P* < 0.05, ^▽▽^ *P* < 0.01  *versus* 10 *μ*g/mL of mmLDL for 12 h. The data are presented as the mean ± SEM. *n* = 8 coronary arteries, from that number of animals.

The Krebs solution containing 63.5 mM K^+^ was used to examine the viability and contractility of the arteries during organ culture. There was no significant difference in the *E*_max⁡_ of the contractile responses induced by K^+^ among the groups (i.e., freshly isolated: 1.90 ± 0.11 mN, organ-cultured: 2.12 ± 0.17 mN, organ-cultured in the presence of mmLDL: 2.05 ± 0.13 mN, organ-cultured in the presence of LDL: 1.98 ± 0.15 mN, *n* = 8, *P* > 0.05). ET-1 induced concentration-dependent contractions in freshly isolated coronary arteries. After 24 h of culture, the ET-1-induced concentration-contraction curve was not significantly different from that of freshly isolated coronary arteries. Culturing for 24 h with mmLDL at 5, 10, or 20 *μ*g/mL shifted the contractile curves induced by ET-1 toward the left in a concentration-dependent manner (Figure 1(a)). The *E*_max⁡_ of the 10 and 20 *μ*g/mL mmLDL groups was increased to 228% ± 20% and 257% ± 23% compared to the control group value (*P* > 0.05). After organ culture for 6 h with 10 *μ*g/mL mmLDL, the ET-1-induced concentration-contractile curve was not significantly affected. Culturing with 10 *μ*g/mL mmLDL for 12, 24, or 48 h shifted the contractile curves induced by ET-1 toward to the left in a time-dependent manner (Figure 1(b)). The *E*_max⁡_ of 24 h mmLDL-supplemented cultures (228% ± 20%) was significantly higher than that of 12 h mmLDL-supplemented cultures (151% ± 15%, *P* < 0.01) but not significantly lower than that of 48 h mmLDL-supplemented cultures (261% ± 23%, *P* > 0.05). mmLDL was used at a concentration of 10 *μ*g/mL with one time point, for 24 h, in the present study. Organ culture for 24 h *per se* did not increase the contractile responses of the arterial segments to ET-1, which could be obviously enhanced by exposure to 10 *μ*g/mL mmLDL. However, 10 *μ*g/mL native LDL did not affect the concentration-contractile curves of coronary artery segments induced by ET-1 (Figure 1(a)).

**Figure 2:**
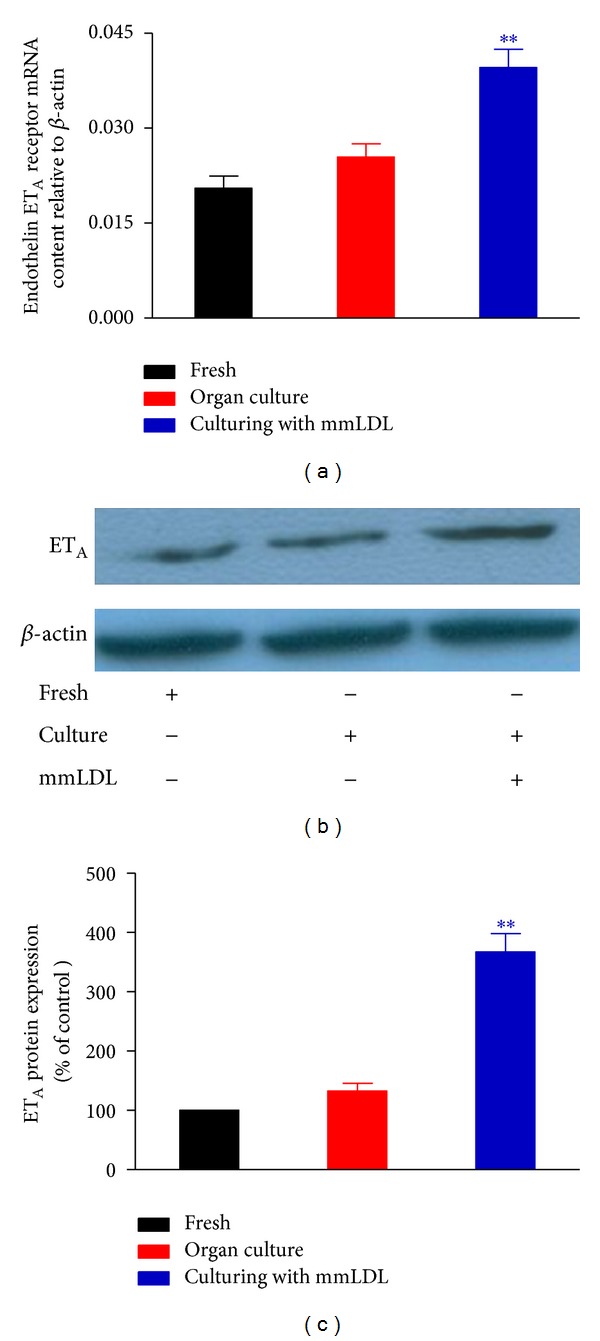
Culturing with mmLDL-induced increase of the level of expression of ET_A_ receptor mRNA ((a) *n* = 5-6 coronary arteries, from that number of animals) and protein ((b and c) *n* = 4 samples, each sample being a pool of 4 coronary arteries). The data are presented as the mean ± SEM. **P < 0.01  *versus* organ culture.

The levels of expression of ET_A_ receptor mRNA and protein in coronary artery segments were determined using real-time PCR and western blotting, respectively. Organ culture did not elevate the mRNA and protein levels of the ET_A_ receptor compared to those of freshly isolated coronary artery segments. Culturing with mmLDL significantly elevated the levels of ET_A_ receptor mRNA and protein compared to those of the control cultures (Figure 2). 

### 3.2. Effect of a PKC Inhibitor on the mmLDL-Induced Upregulation

**Figure 3:**
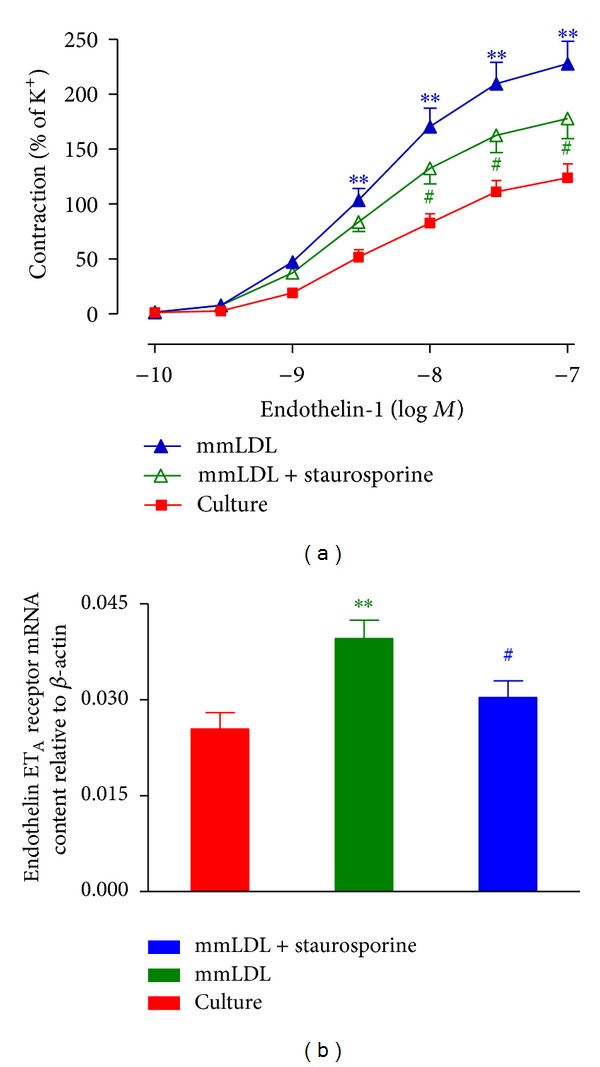
Effect of a PKC inhibitor on the mmLDL induced increase in contractile function and mRNA levels of ET_A_ receptor in the rat coronary artery. After the coronary artery rings were cultured for 24 h with mmLDL (10 *μ*g/mL) in the presence of the PKC inhibitor staurosporine (0.1 *μ*M), the concentration-contraction curves of the rings mediated by ET_A_ receptor ((a) *n* = 8 coronary arteries, from that number of animals) and the levels of the ET_A_ receptor mRNA ((b) *n* = 5-6 coronary arteries, from that number of animals) were determined. Staurosporine inhibited the mmLDL-induced increase in ET_A_ receptor contractile function and mRNA expression. The data are presented as the mean ± SEM. **P < 0.01  *versus* culture, ^#^ *P* < 0.05, *versus* mmLDL.

The presence of staurosporine, a specific inhibitor of PKC, markedly inhibited the mmLDL-induced enhancement of the contractile response to ET-1 and decreased the *E*_max⁡_ from 228% ± 20% in the mmLDL-supplemented group to 178% ±18% (*P* < 0.05) (Figure 3(a), Table 1). In addition, the expression of ET_A_ receptor mRNA and protein in the coronary arterial smooth muscle cells cocultured with staurosporine was lower than that of mmLDL group (Figures 3(b) and 6). 

### 3.3. Effect of MAPK Inhibitors on the mmLDL-Induced Upregulation

**Figure 4:**
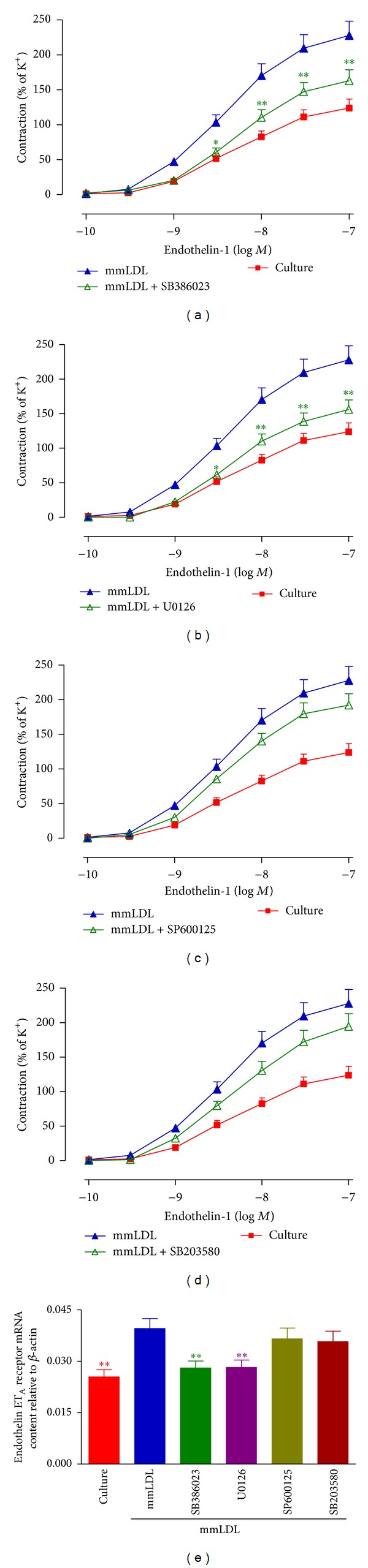
Effect of the MAPK pathway inhibitors on the mmLDL-induced increase in contractile function and levels of ET_A_ receptor mRNA in the coronary artery. After rat coronary arteries were cultured for 24 h with mmLDL (10 *μ*g/mL) in the presence of MAPK inhibitors, including the ERK1/2 inhibitors SB386023 (a), U0126 (b), the JNK inhibitor SP600125 (c), or the p38 inhibitor SB203580 (d), the ET-1-induced concentration-contraction curves were constructed (*n* = 8 coronary arteries, from that number of animals). The effects of the MAPK inhibitors on the level of ET_A_ receptor mRNA are shown ((e) *n* = 5-6 coronary arteries, from that number of animals). The data are presented as the mean ± SEM. *P < 0.05, **P < 0.01  *versus* mmLDL.

After culture for 24 h with mmLDL and specific inhibitors for ERK1/2, the concentration-response curves of ET-1-induced contractions in the SB386023- and U0126-treated groups were markedly shifted toward the right compared to the mmLDL group, in a nonparallel manner (Figures 4(a) and 4(b)). The *E*_max⁡_ and pEC_50_ of contraction in the groups coincubated with mmLDL and SB386023 or U0126 were significantly lower than those of the group incubated with mmLDL (*P* < 0.01 and *P* < 0.05, resp., Table 1). However, the JNK inhibitor SP600125 and the p38 inhibitor SB203580 did not modify the mmLDL effects on the ET-1-induced responses (*P* > 0.05) (Figures 4(c) and 4(d); Table 1). The levels of expression of ET_A_ receptor mRNA and protein in the vascular smooth muscle cells were determined. The results showed that the ERK1/2 inhibitors SB386023 and U0126 significantly attenuated the mmLDL-induced increase of the expression of ET_A_ receptor mRNA and protein. This was paralleled by the decreased ET_A_ receptor-mediated contraction. However, inhibition of JNK and p38 MAPK did not have these effects (Figures 4(e) and 6).

### 3.4. Effect of Inhibition of NF-*κ*B on the mmLDL-Induced Upregulation of ET_A_ Receptors

**Figure 5:**
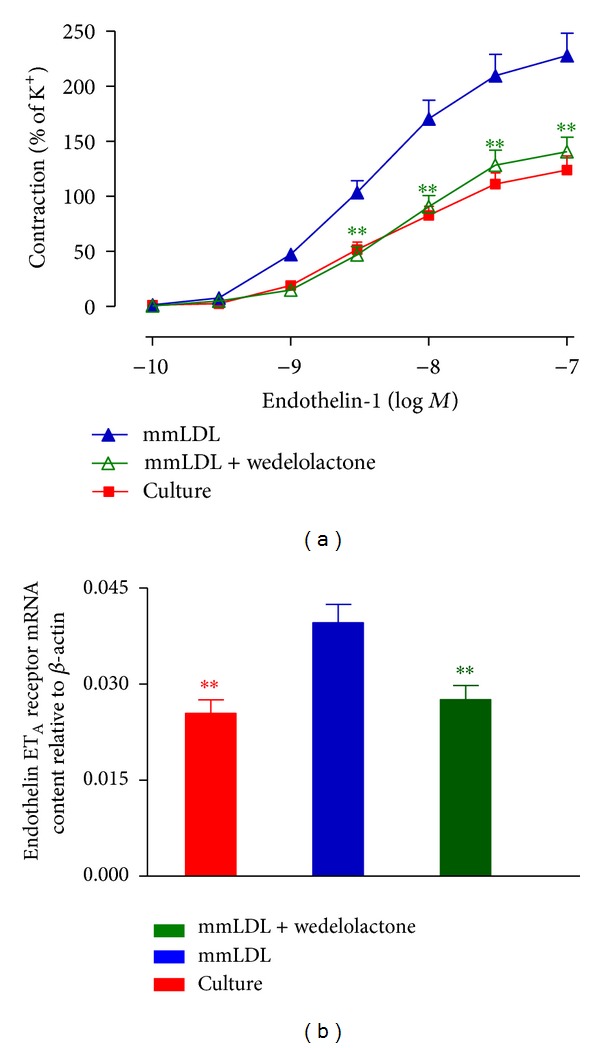
Effect of the NF-*κ*B pathway inhibitor on the mmLDL-induced upregulation of ET_A_ receptors in the coronary artery. Rat coronary artery segments were cultured with mmLDL (10 *μ*g/mL) in the presence of the NF-*κ*B inhibitor wedelolactone (10 *μ*M) for 24 h. The concentration-contraction curves mediated by the ET_A_ receptor ((a) *n* = 8 coronary arteries, the number of animals) and the levels of ET_A_ receptor mRNA ((b) *n* = 6 coronary arteries, from that number of animals) are shown. The data are presented as the mean ± SEM. **P < 0.01  *versus* mmLDL.

**Figure 6:**
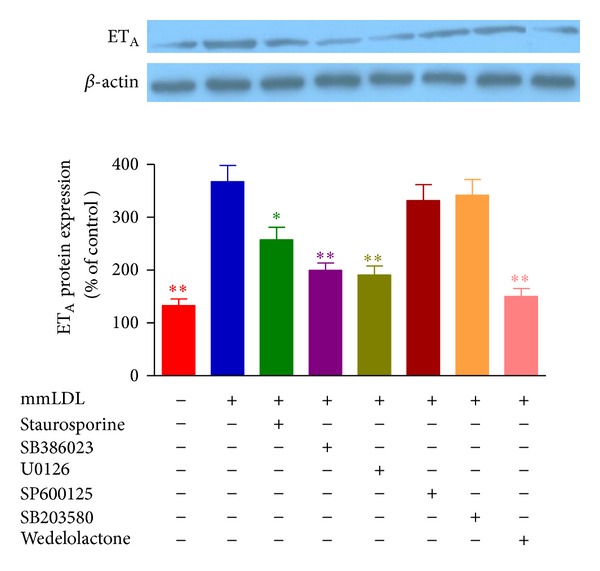
Effect of mmLDL and the intracellular signaling inhibitors on the level of expression of ET_A_ receptor protein in the coronary artery. Rat coronary arteries were cultured with mmLDL (10 *μ*g/mL) in the presence of the PKC inhibitor staurosporine, ERK1/2 inhibitors SB386023 and U0126, JNK inhibitor SP600125, p38 inhibitor SB203580, and NF-*κ*B inhibitor wedelolactone for 24 h. The levels of ET_A_ receptor protein were determined by western blotting. The results are expressed as the mean ± SEM. *n* = 3-4 (each sample being a pool of 4 coronary arteries). *P < 0.05, **P < 0.01  *versus *mmLDL.

A specific inhibitor of the NF-*κ*B pathway, wedelolactone, shifted the mmLDL-enhanced concentration-contraction curve of the coronary artery induced by ET-1 treatment toward the right, with significantly decreased *E*_max⁡_ and pEC_50_ (*P* < 0.01) (Figure 5(a); Table 1). The levels of ET_A_ receptor mRNA and protein in the coronary artery samples showed that wedelolactone significantly inhibited the mmLDL-enhanced expression of the ET_A_ receptor (Figures 5(b) and 6).

## 4. Discussion

ET-1 is the strongest known vasoconstrictor. The upregulation of the ET_A_ receptor leads to enhanced contraction and reduced blood flow, which exacerbates inflammation and contributes to ischemic disease [14, 30, 31]. The present work has contributed to the elucidation of the intracellular signal transduction pathways involved in the mmLDL-induced regulation of the ET_A_ receptor. Culturing rat coronary arteries with mmLDL resulted in the upregulation of ET_A_ receptor-mediated contraction. Likewise, the ET_A_ receptor immunostaining intensity and mRNA levels were increased. The inhibition experiments revealed that the PKC and ERK1/2 MAPK pathways and the downstream NF-*κ*B transcriptional factor signaling pathway were involved in the mmLDL-mediated process of upregulating the ET_A_ receptor.

Previous studies used organ cultures of coronary arteries and other vessels as an experimental model for the detailed delineation of the regulation of endothelin receptors because the changes that occur in this model are similar to those frequently observed in cardiovascular disease. This is the first time that culturing rat coronary arteries in the presence of mmLDL was evaluated as an experimental model for the regulation of the ET_A_ receptor. Culturing rat coronary artery segments with mmLDL for 24 h resulted in increased ET-1-induced contraction which was mediated by the ET_A_ receptor. Furthermore, the real-time PCR results demonstrated that mmLDL treatment elevated the level of ET_A_ receptor mRNA, and western blot analysis showed that the mmLDL treatment increased the expression of ET_A_ receptor protein. The present study showed that organ culture *per se* did not affect the contractile response mediated by the ET_A_ receptor, which agrees well with the results of the previous study [22]. LDL treatment did not significantly increase the contractility of coronary arterial segments, suggesting that LDL might not affect the regulation of the ET_A_ receptor. Taken together, these results suggest that the mmLDL-supplemented organ culture model is suitable for stimulating the upregulation of the ET_A_ receptor in rat coronary arteries. The changes that occur during culture with mmLDL might be comparable to those that occur in cardiovascular disease. Studies using porcine models found large numbers of ET_A_ receptors in the tunica media and neointima of porcine saphenous vein grafts [32]. *In vivo*, secondhand smoke increased the contractile response of the mouse airway to ET-1 [33]. Exposure to secondhand smoke also upregulated the level of ET_A_ receptors in rat cerebral and coronary arteries via the Raf/ERK/MAPK pathway [23, 24]. In human studies, the ET_A_ receptors are localized at the regions of the saphenous vein where cellular proliferation occurs [34]. The upregulation of contractile response receptors in arterial smooth muscle cells is observed in ischemic vascular diseases [35, 36], and suppressing receptor upregulation or blocking the receptors has been shown to be beneficial in the case of vascular damage [37, 38]. Thus, upregulation of these receptors is a key event in the development of vascular diseases. 

There is a significant relationship between receptor upregulation and its stimulatory factors. This study aimed to elucidate the role of the PKC and MAPK intracellular signal transduction pathways and the downstream NF-*κ*B transcriptional factor signaling pathway in the mmLDL-induced upregulation of the ET_A_ receptor. PKC takes part in signal transduction events in response to certain stimuli. PKC has been reported to be activated by ET-1 in diabetic vascular smooth muscle cells and to increase extracellular matrix deposition, cellular hypertrophy, and cell proliferation [39]. In the present experiments, inhibition of PKC by staurosporine reduced the mmLDL-enhanced contraction mediated by the ET_A_ receptor and the mmLDL-induced increase of the ET_A_ receptor mRNA and protein levels. This result suggests that the PKC pathway is involved in the process of upregulating the ET_A_ receptor in coronary arteries. This suggestion is supported by studies in rats that demonstrated that upregulation of the endothelin receptors involves PKC [40–42]. Furthermore, *in vivo* rat studies demonstrated that PKC inhibitors prevent the upregulation of vascular endothelin receptors and reverse the reduction of cerebral blood flow subsequent to subarachnoid hemorrhage [43]. PKC has previously been reported to contribute to the vascular remodeling that occurs during hypertension [44]. In addition, PKC has been implicated in the induction of hypertrophy of cardiomyocytes, and PKC activation has also been shown to aggravate hypoxic myocardial injury and to be proarrhythmic [45]. 

PKC activates the MEK/ERK pathways at several levels [46]. U0126, a noncompetitive inhibitor of the MEK substrates [47], blocks the enzymatic activity of MEK1/2 and subsequently inhibits the activation of ERK1/2. SB386023 inhibits the MAPKKK upstream of MEK, namely, the Raf family [48]. Raf binds to and activates MEK and no other MAPKK, which makes it specific for the ERK pathway [49]. Previous studies have shown that ERK1/2 is involved in the upregulation of the ET receptors that mediate the contraction of the rat cerebral artery, coronary artery and superior mesenteric artery [18, 19, 22, 23, 28]. In the present study, both of the ERK1/2 inhibitors used, U0126 and SB386023, significantly decreased the mmLDL-enhanced contraction mediated by the ET_A_ receptor and significantly attenuated the mmLDL-induced increase of the ET_A_ receptor mRNA and protein levels. These results indicate that the ERK1/2 pathway is involved in the mmLDL-induced upregulation of the ET_A_ receptor in coronary arteries. ERKs mediate cellular responses that are initiated by growth factors [50] and have been implicated in cardiovascular and cerebrovascular disease. Inhibition of ERK1/2 attenuates the lipoprotein (*α*)-induced growth of human vascular smooth muscle cells, which is an independent risk factor for cardiovascular disease [51]. *In vivo* studies have shown that ERK1/2 inhibitors prevent the upregulation of the vascular ET_B_ receptor and reverse the reduction of cerebral blood flow after subarachnoid hemorrhage in rats [52].

Three different JNK pathways (JNK1, -2, and -3) have been identified in humans. JNK1 and JNK2 have a broad tissue distribution, whereas JNK3 is primarily localized in neuronal tissues and cardiac myocytes. SP600125 inhibits the JNK1, -2, and -3 pathways [53]. The JNK inhibitor SP600125 and the p38 inhibitor SB203580 had no obvious effect on the mmLDL-induced increase of contractile function and the expression of ET_A_ receptor mRNA and protein. These results suggested that the JNK and p38 MAPK signaling pathways might not be involved in the upregulation of ET_A_ receptors induced by mmLDL. In previous studies, p38 MAPK was found to not be involved in the ET_B_ receptor-mediated elevation of the contractility of organ-cultured rat middle cerebral arteries and porcine coronary arteries [54, 55], and we also demonstrated that the p38 MAPK pathway was not involved in the mmLDL-induced upregulation of the ET_B_ receptor in the rat coronary artery [18]. It was reported that the JNK pathway is not involved in either the mmLDL-induced or the DSP-induced elevation of vascular contraction or the expression of the ET_B_ receptor of the rat coronary artery and basilar artery [18, 22]. 

Wedelolactone, an inhibitor of the NF-*κ*B signal transduction pathway, prevents the phosphorylation and degradation of I*κ*B, blocking the translocation of NF-*κ*B to the nucleus. The present study showed that the ET_A_ receptor-mediated vascular contraction was upregulated during culture in the presence of mmLDL. Moreover, the real-time PCR and western blotting analyses showed ET_A_ receptor-mediated upregulation of the levels of expression of ET_A_ receptor mRNA and protein. The results suggest that the mmLDL-induced alteration in ET_A_ receptor expression involves increased transcription. Treatment with wedelolactone almost abolished the mmLDL-induced increase in the ET_A_ receptor-mediated function and ET_A_ receptor expression in the coronary artery. These results strongly suggested that the NF-*κ*B pathway is involved in the mmLDL-induced upregulation of the ET_A_ receptor. This result is consistent with the previous finding that NF-*κ*B appears to be involved in the upregulation of the level of the ET_B_ receptor [18, 19, 22, 25]. 

mmLDL is a risk factor for coronary artery disease. Recent results obtained by our group showed that the increased endothelin-induced contraction in organ cultures containing mmLDL could be attributed to the upregulation of endothelin receptors on the vascular smooth muscle cells. Using organ cultures of cerebral arteries in mmLDL-supplemented solutions, we demonstrated that the expression of the ET_B_ receptor was upregulated in the vascular smooth muscle cells and that PKC, MAPK, and NF-*κ*B were involved in the intracellular mechanisms leading to this upregulation [19]. mmLDL also upregulates the level of ET_B_ receptors in coronary arteries by activating ERK1/2 MAPK and the NF-*κ*B transcription factor [18]. In the present work, we demonstrated that culturing coronary arteries with mmLDL increased their ET-1-induced contraction in a concentration-dependent and time-dependent manner and increased the expression of ET_A_ receptor mRNA and protein. The activation of the upstream intracellular PKC and ERK1/2 MAPK pathways and the downstream NF-*κ*B inflammatory signaling pathway is mainly responsible for this upregulation.

In conclusion, mmLDL induces an upregulation of ET_A_ receptors in coronary artery, which may contribute to the development of ischemic cardiovascular diseases. The molecular mechanisms involve the activation of PKC and ERK1/2 MAPK pathways and the downstream NF-*κ*B signaling pathways. Understanding the upregulation and underlying molecular mechanisms may lead to novel treatments of cardiovascular disease.

## Figures and Tables

**Table 1:** Contractile effects of endothelin-1 (ET-1) in coronary artery.

	*n*	Endothelin-1	
		*E*_max⁡_ (%)	pEC_50_
Fresh	8	100 ± 10**	7.88 ± 0.10**
24 h culture	8	124 ± 13**	8.04 ± 0.09**
24 h culture + mmLDL	8	228 ± 20	8.46 ± 0.08
mmLDL + staurosporine	8	178 ± 18*	8.11 ± 0.10*
mmLDL + SB386023	8	163 ± 15**	7.92 ± 0.09**
mmLDL + U0126	8	156 ± 14**	8.04 ± 0.11*
mmLDL + SP600125	8	192 ± 16	8.37 ± 0.12
mmLDL + SB203580	8	194 ± 18	8.29 ± 0.10
mmLDL + wedelolactone	8	141 ± 13**	7.96 ± 0.09**

Responses to endothelin-1 are expressed as *E*_max_ in percent of 63.5 mM K^+^-induced contraction and in pEC_50_ values (negative logarithm of the molar concentration that produces half-maximum contraction). The data are expressed as mean ± SEM. Statistical analyses were performed using two-way ANOVA followed by Dunnett's test and Student's *t*-test with Welch's correction. *n* = number of animals examined in rats. *P < 0.05, **P < 0.01 versus 24 h culture + mmLDL.

## Abbreviations

ERK1/2: Extracellular signal-regulated proteins 1 and 2
ET-1: Endothelin-1
ET_A_: Endothelin type A
MAPK: Mitogen-activated protein kinase
mmLDL: Minimally modified low-density lipoprotein
oxLDL: Oxidized low-density lipoprotein
PKC: Protein kinase C.
